# Clinical features of patients with homozygous complement *C4A* or *C4B* deficiency

**DOI:** 10.1371/journal.pone.0199305

**Published:** 2018-06-21

**Authors:** Inka Liesmaa, Riitta Paakkanen, Asko Järvinen, Ville Valtonen, Marja-Liisa Lokki

**Affiliations:** 1 Division of Infectious Diseases, Inflammation Center, University of Helsinki and Helsinki University Hospital, Helsinki, Finland; 2 Transplantation Laboratory, Medicum, University of Helsinki, Helsinki, Finland; 3 Division of Cardiology, Department of Medicine, University of Helsinki and Helsinki University Hospital, Helsinki, Finland; Institut d'Investigacions Biomediques de Barcelona, SPAIN

## Abstract

**Introduction:**

Homozygous deficiencies of complement *C4A* or *C4B* are detected in 1–10% of populations. In genome-wide association studies *C4* deficiencies are missed because the genetic variation of *C4* is complex. There are no studies where the clinical presentation of these patients is analyzed. This study was aimed to characterize the clinical features of patients with homozygous *C4A* or *C4B* deficiency.

**Material and methods:**

Thirty-two patients with no functional *C4A*, 87 patients with no *C4B* and 120 with normal amount of *C4* genes were included. *C4A* and *C4B* numbers were assessed with genomic quantitative real-time PCR. Medical history was studied retrospectively from patients’ files.

**Results:**

Novel associations between homozygous *C4A* deficiency and lymphoma, coeliac disease and sarcoidosis were detected. These conditions were present in 12.5%, (4/32 in patients vs. 0.8%, 1/120, in controls, OR = 17.00, 95%CI = 1.83–158.04, p = 0.007), 12.5% (4/32 in patients vs. 0%, 0/120 in controls, OR = 1.14, 95%CI = 1.00–1.30, p = 0.002) and 12.5%, respectively (4/32 in patients vs. 2.5%, 3/120 in controls, OR = 5.571, 95%CI = 1.79–2.32, p = 0.036). In addition, *C4A* and *C4B* deficiencies were both associated with adverse drug reactions leading to drug discontinuation (34.4%, 11/32 in *C4A-*deficient patients vs. 14.2%, 17/120 in controls, OR = 3.174, 95%CI = 1.30–7.74, p = 0.009 and 28.7%, 25/87 in *C4B-*deficient patients, OR = 2.44, 95%CI = 1.22–4.88, p = 0.010).

**Conclusion:**

This reported cohort of homozygous deficiencies of *C4A* or *C4B* suggests that *C4* deficiencies may have various unrecorded disease associations. *C4* gene should be considered as a candidate gene in studying these selected disease associations.

## Introduction

The complement system is an essential humoral defence mechanism that is involved in maintaining tissue homeostasis, innate immune reactions, activation and propagation of adaptive immune reactions as well as in non-immune functions such as lipid metabolism, synapsis maturation and blood coagulation. The complement cascade can be activated by three pathways; classical, alternative or lectin pathway. To maintain these varied functions, the complement system is meticulously regulated. Dysfunctions in the complement system have been linked with risk of various infections, autoimmune conditions such as systemic lupus, rheumatoid arthritis and asthma, as well as sepsis, ischemia-reperfusion injury and age-related macular degeneration [1].

The complement component C4 plays a role in the activation of classic and lectin pathways, leading to cleavage of C2, C3 and C5. The C4 protein is encoded by two slightly different adjacent genetic loci, *C4A* and *C4B*, located within the major histocompatibility complex (MHC) class III region on the short arm of chromosome 6 (MIM +120810 and *120820, respectively) [2–4]. Consequently, there are both C4A and C4B proteins circulating in the blood and tissues, which together form the commonly measured C4 concentration. Most individuals have four C4 genes. Approximately 60% of healthy individuals have two C4A and two C4B genes [5, 6]. In the Finnish population, 59% individuals have one *C4A* and one *C4B* gene in a chromosome [7]. However, the total number of C4 genes may vary between 2 and 8 [8].

The presence of no functional *C4A* or *C4B* genes causes complete *C4A* or *C4B* deficiency and is called homozygous C4 deficiency. The presence of one *C4A* or *C4B* gene is called heterozygous *C4A* or *C4B* deficiency [8]. Compared to deficiencies of other complement components of the classical pathway, homozygous *C4A* and *C4B* deficiencies are rather common [9]. Homozygous *C4A* deficiency is detected in 1–6% and homozygous *C4B* deficiency in 1–10% of studied healthy populations [7, 10–15]. Heterozygous *C4* deficiency is more common; 12–21% of healthy populations are heterozygously *C4A*-deficient and 20–41% of the populations have a heterozygous deficiency of *C4B* [8, 10, 11, 13–15]. Deficiency of all *C4* genes (neither functional *C4A* nor *C4B* genes present) is extremely rare and to date, only 28 patients have been described in the literature [9, 16, 17]. Patients with no functional *C4* genes have been reported to have SLE (n = 17), SLE-like disease (n = 5), kidney diseases (n = 6), and repeated or invasive infections (n = 7) [9].

In disease association studies, homozygous and heterozygous *C4A* or *C4B* deficiencies are usually grouped together under the general term of “*C4A* or *C4B* deficiency”. The studies distinguishing homozygous *C4* deficiencies have repeatedly reported association between homozygous *C4A* deficiency and systemic lupus erythematosus (SLE)[10, 18–20]. There is also one study reporting association with pulmonary tuberculosis [21] and one with capillary leak syndrome during cardiopulmonary bypass in children [22]. Homozygous *C4B* deficiency has been associated with coronary artery disease [23], glomerular disease and infections in case reports [24, 25]. However, the number of cases in these reports is very low, ranging from three to twenty-six.

This study was aimed to systematically assess the clinical features and characteristics as well as disease associations of patients with either homozygous *C4A* or *C4B* deficiency. We detected novel, previously unrecorded links with disease conditions and confirmed previously known associations.

## Patients and methods

### Samples

Individuals from Helsinki University Hospital with homozygous *C4A* (n = 32) or *C4B* (n = 87) deficiency were identified from our Laboratory’s database between years 2004 and 2011 by screening 2173 individuals. Randomly selected individuals with two functional *C4A* and *C4B* genes (n = 120) from the same record served as controls. Cases with two *C4A* and *C4B* genes were chosen as controls as this is the most common genetic combination of the background population and in order to make the control population as homogenous as possible to ease the comparisons between cases and controls. The controls were selected over the same time range as the first and middle sample of a given month. The Local Ethics Committee’s approval was not needed due to anonymous register-like nature of the study.

### Medical history

The medical records were retrospectively evaluated for diagnosed clinical diseases and descriptive symptoms. The medical history and diagnoses were individually retrieved as defined by the treating clinician. The searched diagnoses/conditions are listed below.

Autoimmune conditions: SLE, rheumatoid arthritis, spondylarthritis ancylopoetica, seronegative spondylarthropathy (SSA), type I diabetes mellitus, coeliac disease, autoimmune gastritis, primary biliary cirrhosis, autoimmune hepatitis, autoimmune haemolytic anaemia, autoimmune neutropenia, idiopathic thrombocytopenic purpura, glomerulonephritis, IgA nephropathy and asthma.

Infections were categorized as 1) invasive infections including bacteremia, pneumonia and meningitis, 2) recurrent central nervous system Herpes simplex virus (HSV) manifestations, and 3) recurrent tonsillitis or sinusitis which required operative treatment.

Neurological diagnoses and signs: Epilepsy, multiple sclerosis, vertigo, migraine or severe headache, spasticity or ataxia of unknown causes, numbness, myalgia, paresthesias and paralyses of unknown causes.

Gastrointestinal diseases and signs: inflammatory bowel disease, abdominal pain necessitating hospital investigations, irritable bowel syndrome, and recurrent *Clostridium difficile* colitis.

Other symptoms: reactive arthritis, hypothyroidism.

Adverse drug effects: Any mention of adverse drug reactions leading to drug discontinuation (and avoidance), toxic reactions and side effects were recorded.

Allergies: Reported by patients included mainly pollen and/or animal epithelium, nickel and iodine.

Psychological conditions: Psychosis, schizophrenia, severe depression and anxiety.

### Gene analyses

The copy numbers of *C4A*, *C4B* and the most common silencing mutation of *C4A*, *CTins*, were recorded with a validated real-time quantitative polymerase chain reaction as described in detail elsewhere [7]. In brief, genomic DNA was amplified with SYBR® Green labelling using isotype-specific primers (Rotor-Gene 3000, Qiagen, Austria). The resulting fluorescence curve was analysed in relation to controls and housekeeping gene fluorescence recordings (Rotor-Gene, software v 6.0, Qiagen). The number of functional *C4A* was determined by reducing the number of *CTins* copies from the total number of *C4A*. Besides the *CTins*, no other *C4* mutations were screened,.

Human leucocyte antigen (HLA) alleles (HLAB-27*,-35*, HLA-DRB1*01, *03, *04, *15) were characterized with similar quantitative real-time PCR procedure using allele-specific primers and performed in our HLA-laboratory having accreditation by the European Federation for Immunogenetics (EFI). HLA-genotyping data was available from 50% (16/32) of *C4A*-deficient patients, 51% (44/87) of *C4B*-deficient patients and 46% (55/120) of the controls. The HLA data was available if the treating physician had requested for these assays based on clinical judgement.

### Laboratory parameters

Immunoglobulin levels (IgG, M, A and E) and antibody levels against *Herpes simplex* viruses (HSV, types 1 and 2), *Varicella zoster* virus (VZV), nuclear antigens (ANA), neutrophilic cytoplasmic components (ANCA) and rheumatic factor (RF) were collected from medical reports. An abnormal value was indicated if differed from reference values (6.8–15 g/l for IgG, 0.36–2.59 g/l for IgM, 0.88–2.84 g/l for IgA, <1 g/l for IgE, 0.71–1.41 g/l for C3, 0.15–0.5 g/l for C4, positive titre recording (95^th^ percentile) for HSV or VZV, 1:320 for ANA and 1:20 for ANCA, all positive titres represented about 95th percentile level in Finnish population). All laboratory recordings were done by the standard procedures of the accredited diagnostic laboratory of Helsinki and Uusimaa Hospital District (HUSLAB).

### Statistical analyses

Statistical analyses, Chi-square, Student’s t- and Mann–Whitney U test, were performed when appropriate using PASW statistics (v. 20.0). The continuous variables that were normally distributed were also tested with Student’s t-test. Two-tailed p-value <0.05 was considered statistically significant. Odd’s ratios (OR) and their 95% confidence intervals (95%CI) were assessed with Chi-square statistics. The number of autoimmune conditions was assessed with Linear-by-linear association. Sex, decreased complement activity of mannan binding lectin pathway, genetic backgrounds of *C4* deficiencies and the simultaneous presence of HLA-B*35 and HLA-DRB1*01 were studied in subgroup analyses of *C4A* or *C4B* deficiency and compared with the same subgroups in controls. In most cases, all available laboratory parameters were included except for complement activity analyses, where the cases were excluded listwise.

## Results

Age- and sex distributions were similar in all groups. The majority (>75%) of the samples were from Helsinki University Hospital, Division of Infectious Diseases, corresponding to our Laboratory’s general distribution (Table 1).

**Table 1:** Baseline characteristics.

	Homozygous C4A deficiency (n = 32)	Homozygous C4B deficiency (n = 87)	Controls (n = 120)		
	n (%) or mean (SD)	n (%) or mean (SD)	n (%) or mean (SD)	P = (C4A-deficient vs. Controls)	P = (C4B-deficient vs. Controls)
**Age (years)**	45.25 (18.0)	44.08 (14.6)	43.92 (15.2)	0.778	0.900
**Females**	22 (68.8)	59 (67.8)	72 (60.0)	0.365	0.181
**Samples ordered from**				0.036	0.615
Infectious diseases	25 (78.2)	75 (86.2)	108 (90.0)		
Internal medicine	1 (3.1)	7 (8.0)	6 (5.0)		
Other departments or private sector	6 (18.1)	5 (5.7)	6 (5.0)		
**Complement values** ****					
Plasma C3 g/l	1.19 (0.4)	1.11 (0.34)	1.21 (0.4)	0.263	0.025
Low C3 (%)	2 (6.9)	7 (8.5)	4 (3.6)	0.604	0.209
Plasma C4 g/l	0.17 (0.1)	0.2 (0.1)	0.27 (0.1)	<0.0001	<0.0001
Low C4 (%)	5 (17.2)	15 (18.5)	2 (1.8)	0.005	0.0001
Abnormal immunoglobulin levels****					
IgG (%)	10 (35.7)	17 (20.7)	19 (17.0)	0.029*	0.461
IgM (%)	8 (28.6)	11 (13.3)	11 (9.9)	0.026**	0.436
IgA (%)	6 (21.4)	11 (13.4)	13 (11.7)	0.218	0.682
**Positive autoimmune antibodies**					
ANA or ANCA (%)	10 (33.3)	14 (17.5)	17 (16.5)	0.044***	0.836

Data is presented as median (SD) for age, plasma C3, plasma C4 and as n (%) for other variables.C4A, complement component C4A; C4B, Complement component C4B; C3, complement component 3; Abnormal immunoglobulin levels, either elevated or lowered; IgG, Immunoglobulin class G; IgM, Immunoglobulin class M; IgA Immunoglobulin class A; ANA, anti-nuclear antibodies; ANCA, anti-neutrophilic cytoplasmic antibodies.

* OR = 2.719, 95%CI = 1.087–6.804

** OR = 3.636, 95%CI = 1.299–10.181

*** OR = 2.53, 95%CI = 1.00–6.35

**** For cut-offs for abnormal C3, C4, IgG, IgM and IgA leves, see text for Laboratory Parameters

Both patient groups with homozygous *C4A* or *C4B* deficiency had lower blood C4 levels (Table 1). Patients with homozygous *C4A* deficiency had autoantibodies more often than controls (33.3%, 10/32 vs. 16.5%, 17/120, p = 0.044 for positive ANCA or ANA autoantibodies, Table 1). In addition, the IgG and IgM immunoglobulin levels seemed to be more often abnormal (elevated or lowered) among patients with homozygous *C4A* deficiency as compared to controls. Patients with homozygous *C4B* deficiency were more often recorded to have statistically lower complement C3 activation values (Table 1), but the frequency of abnormally low C3 (i.e. below the laboratory reference range) was similar in all groups (data not shown).

### Autoimmune manifestations

Homozygous *C4A* deficiency was associated with increased number of autoimmune conditions (p = 0.047, Table 2). The specific autoimmune conditions were SLE (18.8%, 6/32 vs. 5.8%, 7/120 in *C4A* deficient patients and controls, respectively, OR = 3.75, 95%CI = 1.16–12.01, p = 0.031) and coeliac disease (12.5%, 4/32 vs. 0%, 0/120 in *C4A* deficient patients and controls, p = 0.002).

**Table 2:** Autoimmune conditions in patients with homozygous C4A or C4B deficiency and in patients without C4 deficiencies.

	Homozygous C4A deficiency (n = 32)	Homozygous C4B deficiency (n = 87)	Controls (n = 120)		
	n (%)	n (%)	n (%)	P = (C4A-deficient vs. controls)	P = (C4B-deficient vs. controls)
**Any autoimmune condition**	21 (65.6)	42 (48.3)	55 (45.8)	0.047*	0.728
**Systemic Lupus Erythematosus**	6 (18.8)	8 (9.20)	7 (5.8)	0.031**	0.357
**Coeliac disease**	4 (12.5)	0 (0)	0	0.002***	1.000
**Hypothyreoidism**	2 (6.3)	6 (6.9)	10 (8.3)	1.000	0.702
**Seronegative spondyloarthropathy**	3 (9.4)	7 (8.0)	4 (3.3)	0.162	0.208
**Post-infective prolonged symptoms**	3 (9.4)	12 (13.8)	5 (4.2)	0.241	0.013****
**Reactive arthritis**	1 (3.13)	4 (4.6)	1 (0.8)	0.312	0.164

* OR (95%CI) = 2.26 (1.00–5.09)

** OR (95%CI) = 3.75 (1.16–12.01)

*** OR (95%CI) = 1.14 (1.00–1.30)

**** OR (95%CI) = 3.68 (1.25–10.87)

Homozygous *C4B* deficiency was not associated with autoimmune condition(s). Although there was increased incidence of symptoms detected post-infectiously reminiscent of autoimmune diseases (13.8% vs. 4.17% in *C4B* deficient patients and controls, respectively, OR = 3.68, 95% CI = 1.25–10.87, p = 0.013). These conditions are listed in detail in S1 Table.

### Infections

No differences in rate of recurrent or invasive infections were apparent between patients with homozygous *C4A* or *C4B* deficiency and controls. Central nervous system *Herpes simplex* virus infections were marginally (but not statistically) increased in both *C4* deficient groups (6.3%, 2/32, 5.7%, 5/87 and 1.7% 2/120 in *C4A* deficient, *C4B* deficient and in controls, respectively, data not shown). *HSV type 1* was the causative agent in two *C4A-*deficient and *HSV 2* in five *C4B-*deficient patients.

### Other conditions

In patients with homozygotic *C4A* deficiency, lymphomas were significantly more common than in other groups (12.50%, 4/32 in *C4A* deficient vs. 0.8%, 1/120 in controls OR = 17.00, 95%CI = 1.83–158.04, p = 0.007, Table 3). However, no specific lymphoma type could be identified. In addition, sarcoidosis was more commonly diagnosed in *C4A* deficient patients (12.50%, 4/32 vs. 2.50%, 3/120 OR = 5.571, 95%CI = 1.1789–26.3, p = 0.036, for *C4A* deficient patients and controls, respectively). No differences in frequencies of recorded neurological, ocular or psychiatric diagnoses were apparent.

**Table 3:** Other clinical conditions associated with homozygous C4 deficiencies.

	Homozygous C4A deficiency (n = 32)	Homozygous C4B deficiency (n = 87)	Controls (n = 120)		
	n (%)	n (%)	n (%)	P = (C4A-deficient vs. controls)	P = (C4B-deficient vs. controls)
**Any malignancy**	6 (18.8)	9 (10.3)	8 (6.7)	0.077*	0.442
**Lymphoma**	4 (12.5)	1 (1.1)	1 (0.8)	0.007**	1.000
**Sarcoidosis**	4 (12.5)	4 (4.6)	3 (2.5)	0.036***	0.453
**Ischemia**	2 (6.3)	4 (4.6)	3 (2.5)	0.283	0.394
**Epilepsy**	1 (3.1)	4 (4.6)	4 (3.3)	1.000	0.621
**Migraine**	0 (0.0)	2 (2.3)	3 (2.5)	1.000	1.000
**Psychiatric diagnosis**	0 (0.0)	4 (4.6)	3 (2.5)	1.000	0.453

* OR = 3.231, 95%CI = 1.032–10.115

** OR = 17.00, 95%CI = 1.83–158.04

*** OR = 5.57, 95%CI = 1.18–26.3

### Adverse drug reactions

Patients with homozygous *C4A* or *C4B* deficiency were recorded to have more adverse effects leading to drug discontinuation than controls (34.38%, 11/32 in *C4A* deficient, 28.74%, 25/87 in *C4B* deficient vs. 14.17%, 17/120 in controls, p = 0.009 for *C4A*-deficient vs. controls, p = 0.010 for *C4B*-deficient vs. controls, Table 4). The discontinued medications were most often antibiotics. For *C4A* deficient patients, statistical significance was not attained for any individual category. Patients with *C4B* deficiency, however, had significantly more often discontinued antimicrobial agents than the controls (25%, 22/87 vs. 10%, 12/120, p = 0.003). This difference seemed to be explained by the markedly increased intolerance to sulphonamides (12.8%, 11/87 vs. 0.8%, 1/120, OR = 17.45, 95%CI = 2.21–138.0, p<0.001) and doxycycline (8.1%, 7/87 vs. 0.8%, 1/120, OR = 10.544, 95%CI = 1.27–87.37, p = 0.001 for *C4B*-deficient vs. controls).

**Table 4:** Adverse drug reactions with homozygous C4 deficiency.

	Homozygous C4A deficiency (n = 32)	Homozygous C4B deficiency (n = 87)	Controls (n = 120)		
	n (%)	n (%)	n (%)	P = (C4A-deficient vs. controls)	P = (C4B-deficient vs. controls)
**All adverse drug reactions**	11 (34.4)	25 (28.7)	17 (14.2)	0.009*	0.010**
**Antimicrobial discontinuation**					
Sulfonamides	2 (6.9)	11 (12.8)	1 (0.8)	0.097	0.0003***
Doxycycline	1 (3.5)	7 (8.1)	1 (0.8)	0.352	0.010****
Beta-lactamases	4 (13.8)	11 (12.8)	8 (6.7)	0.250	0.134
Macrolides	0 (0)	6 (7.0)	2 (1.7)	1.000	0.070
Other	0 (0)	4 (4.7)	2 (1.7)	1.000	0.238
Any antimicrobial	6 (20.7)	22 (25.6)	12 (10.0)	0.121	0.003*****
**Anti-inflammatory**					
NSAID	2 (6.9)	3 (3.4)	2 (1.7)	0.195	0.652
ASA	1 (3.5)	5 (5.7)	4 (3.3)	1.000	0.497
Other******					
Any other	0 (0)	8 (9.2)	5 (4.2)	0.585	0.141

NSAID, non-steroidal anti-inflammatory drug; ASA, acetylic salicylic acid

* OR (95%CI) = 3.17 (1.3–7.7)

** OR (95%CI) = 2.44 (1.22–4.88)

*** OR (95%CI) = 17.45 (2.21–137.96)

**** OR (95%CI) = 10.54 (1.27–87.37)

***** OR (95%CI) = 3.09 (1.44–6.67)

****** Other intolerated medicines for *C4B* deficient (barium, alendronate, metamizole + pitophenone, tramadol, antibiotics for tuberculosis, valaciclovir, methylprednisolone). For controls the intolerated medicines (angiotensinogen II inhibitor, beta-blocker, lidocaine and myasthenia combination drugs).

### Complement activity

The activities of complement activation pathways were assessed by the treating clinician in one fourth of the study populations. This number is too small to draw conclusions. The available data is depicted in S2 Table.

### C4 deficiency and remaining C4 genes

The genetic background for homozygous *C4* deficiency was variable (S3 Table). Almost all patients with homozygous *C4A* deficiency had two copies of *C4B* genes (93.75%, 30/32). Most patients with homozygous *C4B* deficiency had three copies of *C4A* genes (68.97%, 60/87) whereas the rest had two *C4A* genes and one patient had four *C4A* genes.

## Discussion

In this relatively large cohort of patients with homozygous *C4A* or *C4B* deficiency, we were able to discover novel disease associations as well as replicate some previously known associations. Where the previous genetic studies of *C4* deficiency have included patients with homozygous and heterozygous *C4* deficiency, this is one of the largest cohorts gathering only patients with homozygous *C4A* or *C4B* deficiency (i.e. no functional *C4A* or *C4B* genes at all). Because the serum levels and the gene numbers do not correlate well, in selecting patients without any *C4A* or *C4B* genes, we are hoping to better clarify disease associations of these conditions.

### Biological effect of homozygous *C4* deficiencies

Although C4A and C4B proteins differ only by 4 amino acids, they exhibit marked differences in chemical reactivity to substrates. Activated C4A protein forms a covalent amide bond with amino groups on peptide antigens, whereas activated C4B binds more efficiently to hydroxyl group containing substrates of carbohydrate antigens [2]. Accordingly, homozygous deficiency of *C4A* has been reported to associate with increased frequency of autoimmune diseases, whereas homozygous *C4B* deficiency has been associated with increased susceptibility of bacterial and enveloped viral infections. However, the reported disease associations of genetic *C4B* deficiency (homozygous and heterozygous deficiency combined) include also common and complex diseases such as psychiatric disorders and atherosclerosis [26–28].

In our study, somewhat surprisingly, the homozygous deficiency of *C4A* or *C4B* were not associated with significantly increased frequency of infectious or autoimmune disease burden (except for the association between SLE and *C4A* deficiency), at least when compared with hospitalized controls. If homozygous *C4A* deficient patients would have significantly aberrant immune clearance, the patients could be expected to exhibit features of aberrant autoimmune reactions, despite the redundancy of the immune system. Correspondingly, the homozygous *C4B* deficient patients would be expected to have at least some kind of signal of increased rate of infections. We could not see such differences, but due to the lack of healthy controls, we were not able to reliably assess the role of infections in this material.

For homozygous *C4A* deficient patients in this study population, the majority of patients had two copies of *C4B*. Thus, *C4B* copy number did not seem to compensate for the lacking *C4A* genes. However, it is not known, to which extent the local production of *C4B* could be increased due to increased demand. In the homozygous *C4B* deficient patients, the majority did have one “extra” copy of *C4A* (three copes in total). Whether this “increase” in *C4A* copies could explain for the small amount of disease associations with homozygous *C4B* deficiency detected in this study remains unknown. In addition, the plasma C4 protein concentration is only partly determined by *C4* gene copy number and is also affected by the size variations as well [29, 30]. Unfortunately, the size variation of *C4* genes and the activities of different complement activation routes were not routinely assessed in this study. In a steady stage, this would naturally be very informative. In addition, a C4-independent activation route of the complement system has been described [31].

As with many other studies assessing the HLA genes, we must account for the surrounding gene region. HLA genes are inherited in tightly connected “haploblocks”, in which the normal laws or genetic recombination do not apply [32, 33]. Without extensive and detailed data or some kind of biological modelling, in mere association studies, the causative nature of the gene marker in question can only be contemplated. Correspondingly, even with the strongest known association between *C4A* deficiency and SLE, the surrounding genetic landscape is playing an essential role in disease susceptibility [10]. In addition, it has recently been shown that despite the linkage with the surrounding genes, *C4* deficiency is not reliably assessed in GWAS [10]. This makes both the disease association studies and analyses more challenging.

### Homozygous *C4A* deficiency

We discovered that patients with homozygous *C4A* deficiency had a significantly increased prevalence of lymphomas, with an OR of 17. To our knowledge, this association has not been previously reported. However, the HLA gene region has been linked with various types of haematological malignancies in multiple studies. Unfortunately, we could not reliably classify the subtypes of these lymphomas and therefore, were unable to further assess the specific disease associations.

It has to be borne in mind that our material consists of relatively small number of patients. In addition, our patients were suffering from difficult or recurrent infections, and the majority was treated in the Department of Infectious Diseases. Even though the control population consisted of similar patients and did not show increased incidence on lymphomas, we cannot speculate, whether the association between homozygous *C4A* deficiency and haematological malignancy could be mediated or underlined by infections the patients were suffering from.

We found the increased incidence of coeliac disease in patients with homozygous *C4A* deficiency. HLA class II alleles are known to confer strong risk for coeliac disease and HLA testing is recommended in exclusion of coeliac disease in certain patients [34]. The *C4A* deficiency has been linked with coeliac disease, most likely due to linkage disequilibrium with the surrounding HLA-alleles [35]. The common ancestral haplotype (AH8.1) has been associated with coeliac disease and various other autoimmune conditions including sarcoidosis [36, 37]. We were able to replicate the finding that the patients with homozygous *C4A* deficiency had almost six-fold more sarcoidosis corresponding to the previous study in Finnish sarcoidosis patients [38]. As with many other disease association studies, based on the data at hand, it is difficult to state, which of the associated gene markers, if any, comprises the true causative factor.

We also were able to confirm the previously reported associations between homozygous *C4A* deficiency and autoimmune condition SLE [10, 18, 19, 20]. Homozygous deficiency of *C4A* is a predisposing factor for SLE, but not all homozygous *C4A* deficient patients develop SLE [10, 18]. The increased incidence of autoantibodies in the homozygous *C4A* deficient patients supports the role of *C4A* deficiency in autoimmunity.

In previous studies in Finland, heterozygous *C4A* deficiency has been shown to associate with recurrent or chronic respiratory tract infections [39]. We were not able to replicate these findings in our material with patients with diseases necessitating hospital treatment. Whether there would be an association if the control material would have consisted of general population, remains unknown.

### Homozygous *C4B* deficiency

In our study, patients with homozygous *C4B* deficiency seemed to have more intolerance to sulphonamides and doxycycline as well as suffering more often from various post-infectious symptoms. The association between drug discontinuation was strong; ORs ranged from 10 to 17. Although the term “post-infectious symptoms” is anything but exact it is an existing and clinically difficult entity that is difficult to verify. The recorded symptoms ranged from vasculitis to prolonged fever, arthralgia and myocarditis after infection. It is impossible to determine the role of infection in these cases but in all of these cases, the symptomology had started soon after bacterial or viral infection and could not be corrected by medical treatment. This corresponds to our previous finding from a patient with persistent arthralgia and myalgia 6 months after acute SINV infection [40].

The data on infection-proneness of *C4* deficiency is controversial [41]. In the previous studies, (mostly homo- and heterozygous deficiency combined) *C4B* deficiency has been linked with increased rate of invasive infections [27]. However, recent association studies have challenged this by inverse associations [21,41–43]. In our cohort, homozygous *C4B* deficient patients did not have increased rate of invasive infections. Due to the biased nature of our data with similar background of controls, we cannot reliably assess the role of homozygous *C4B* deficiency in meningitis or in invasive infections. According to our data, it seems however, that homozygous *C4B* deficiency alone is not significant in causing invasive infections as only a small part of *C4B*-deficient patients suffered from such infections.

### Limitations

The limitations of our study are the relatively small number of homozygous *C4A* deficient patients although this is among the largest clinical studies on homozygous C4 deficiencies, the lack of complete HLA-types and of follow-up data. In addition, another limitation is that the patient data was retrospectively retrieved from medical recordings. We cannot therefore evaluate the medical conditions based on diagnostic criteria.

Due to the rarity of this genetic phenomenon (1–6%), a very large population cohort (1666–10 000) should be screened to attain a population of 100 patients.

In addition, multiple hypotheses in multiple conditions were tested without correcting the p-value, thus the validity of our findings has to be replicated in an independent patient sample. However, as this study is hypothesis-forming rather than hypothesis-confirming, the p-value correction for multiple testing does not need to be stringently followed. Although the background population is highly selected, both patients and controls were drawn from the same cohort. Population- based screening and follow-up studies are needed to determine causality and the applicability to general population. We do not intend to elucidate the disease associations, but merely characterize this population and launch new hypotheses.

### Conclusion

Homozygous *C4A* and *C4B* deficiencies differ in their clinical characteristics and disease associations. In addition to the previous disease associations, we discovered that homozygous *C4A* deficiency might be associated with lymphoma and sarcoidosis and that homozygous *C4B* deficiency might be associated with prolonged post-infectious symptoms and drug discontinuation due to adverse reactions, which occurred mainly with antibiotics. These notions may open novel pathways to assess the role of C4 in these conditions. Therefore, novel and larger studies are warranted.

## Supporting information

S1 Table Post-infectious symptoms in 20 patients with homozygous C4B deficiency. (DOCX)

S2 Table Available complement activities in study populations. (DOCX)

S3 Table Number of C4 genes in the study populations. (DOCX)

## References

[1] Ricklin D, Hajishengallis G, Yang K, Lambris JD. Complement: A key system for immune surveillance and homeostasis. Nat Immunol. 2010;11: 785–797. doi: 10.1038/ni.1923 20720586 10.1038/ni.1923 PMC2924908

[2] Yu CY, Belt KT, Giles CM, Campbell RD, Porter RR. Structural basis of the polymorphism of human complement components C4A and C4B: Gene size, reactivity and antigenicity. EMBO J. 1986;5: 2873–2881. 2431902 10.1002/j.1460-2075.1986.tb04582.x PMC1167237

[3] Yu CY. The complete exon-intron structure of a human complement component C4A gene. DNA sequences, polymorphism, and linkage to the 21-hydroxylase gene. Journal of immunology (Baltimore, Md.: 1950). 1991;146: 1057–1066. 1988494

[4] Dangel AW, Mendoza AR, Menachery CD, Baker BJ, Daniel CM, Carroll MC, et al The dichotomous size variation of human complement C4 genes is mediated by a novel family of endogenous retroviruses, which also establishes species-specific genomic patterns among old world primates. Immunogenetics. 1994;40: 425–436. 7545960 10.1007/BF00177825

[5] Chung EK, Yang Y, Rupert KL, Jones KN, Rennebohm RM, Blanchong CA, et al Determining the one, two, three, or four long and short loci of human complement C4 in a Major Histocompatibility Complex haplotype encoding C4A or C4B proteins. Am J Hum Gene. 2002;71: 810–822. 10.1086/342778 PMC378538 12224044

[6] Chung EK, Wu YL, Yang Y, Zhou B, Yu CY. Human complement components C4A and C4B genetic diversities: Complex genotypes and phenotypes. Curr Protoc Immunol. 2005;Chapter 13: Unit 13 18. 10.1002/0471142735.im1308s68 18432942

[7] Paakkanen R, Vauhkonen H, Eronen KT, Järvinen A, Seppänen M and Lokki M-L. Copy number analysis of complement C4A, C4B and C4A silencing mutation by real-time quantitative polymerase chain reaction. PLoS One. 2012;7: e38813 doi: 10.1371/journal.pone.0038813 22737222 10.1371/journal.pone.0038813 PMC3380926

[8] Blanchong CA, Zhou B, Rupert KL, Chung EK, Jones KN, Sotos JF, et al Deficiencies of human complement component C4A and C4B and heterozygosity in length variants of RP-C4-CYP21-TNX (RCCX) modules in caucasians. The load of RCCX genetic diversity on Major Histocompatibility Complex-associated disease. The Journal of experimental medicine. 2000;191: 2183–2196. 10859342 10.1084/jem.191.12.2183 PMC2193198

[9] Lipsker D, Hauptmann G. Cutaneous manifestations of complement deficiencies. Lupus. 2010;19: 1096–1106. doi: 10.1177/0961203310373370 20693203 10.1177/0961203310373370

[10] Boteva L, Morris DL, Cortes-Hernandez J, Martin J, Vyse TJ and Fernando MM. Genetically determined partial complement C4 deficiency states are not independent risk factors for sle in UK and Spanish populations. Am J Hum Genet. 2012;90: 445–456. doi: 10.1016/j.ajhg.2012.01.012 22387014 10.1016/j.ajhg.2012.01.012 PMC3309188

[11] Seppanen M, Suvilehto J, Lokki ML, Notkola I-L, Järvinen A, Jarva H, et al Immunoglobulins and complement factor C4 in adult rhinosinusitis. Clin Exp Immunol. 2006;145: 219–227. doi: 10.1111/j.1365-2249.2006.03134.x 16879240 10.1111/j.1365-2249.2006.03134.x PMC1809671

[12] Boteva L, Imagen, Wu YL, Cortes-Hernandez J, Martin J, Vyse TJ, et al Determination of the loss of function complement C4 exon 29 CT insertion using a novel paralog-specific assay in healthy UK and Spanish populations. PloS one. 2011;6: e22128 doi: 10.1371/journal.pone.0022128 21857912 10.1371/journal.pone.0022128 PMC3153930

[13] Szilagyi A, Blasko B, Szilassy D, Fust G, Sasvari-Szekely M and Ronai Z. Real-time pcr quantification of human complement C4A and C4 B genes. BMC genetics. 2006;7: 1. 10.1186/1471-2156-7-1 PMC1360677 16403222

[14] Wahrmann M, Dohler B, Ruhenstroth A, Haslacher H, Perkmann T, Exner M, et al Genotypic diversity of complement component C4 does not predict kidney transplant outcome. J Am Soc Nephrol. 2011;22: 367–376. doi: 10.1681/ASN.2010050513 21164027 10.1681/ASN.2010050513 PMC3029909

[15] Wouters D, van Schouwenburg P, van der Horst A, De Boer M, Schooneman D, Kujipers TW, et al High-throughput analysis of the C4 polymorphism by a combination of MLPA and isotype-specific ELISA's. Mol Immunol. 2009;46: 592–600. doi: 10.1016/j.molimm.2008.07.028 19062096 10.1016/j.molimm.2008.07.028

[16] Wu YL, Hauptmann G, Viguier M, Yu CY. Molecular basis of complete complement C4 deficiency in two North-African families with systemic lupus erythematosus. Genes and immunity. 2009;10: 433–445. doi: 10.1038/gene.2009.10 19279649 10.1038/gene.2009.10 PMC2767122

[17] Lokki ML, Circolo A, Ahokas P, Rupert KL, Yu CY and Colten HR. Deficiency of human complement protein C4 due to identical frameshift mutations in the C4A and C4B genes. J Immunol. 1999;162: 3687–3693. 10092831

[18] Yang Y, Chung EK, Wu YL, Savelli SL, Nagaraja HN, Zhou B, et al Gene copy-number variation and associated polymorphisms of complement component C4 in human systemic lupus erythematosus (SLE): Low copy number is a risk factor for and high copy number is a protective factor against SLE susceptibility in European Americans. Am J Hum Genet. 2007;80: 1037–1054. doi: 10.1086/518257 17503323 10.1086/518257 PMC1867093

[19] Ittiprasert W, Kantachuvesiri S, Pavasuthipaisit K, Verasertniyom O, Chaomthum L, Totemchokchyakarn K, et al Complete deficiencies of complement C4A and C4B including 2-bp insertion in codon 1213 are genetic risk factors of systemic lupus erythematosus in thai populations. J Autoimmun. 2005;25: 77–84. doi: 10.1016/j.jaut.2005.04.004 15998580 10.1016/j.jaut.2005.04.004

[20] Man XY, Luo HR, Li XP, Yau YG, Mao CZ and Zhang YP. Polymerase chain reaction based C4Aq0 and C4Bq0 genotyping: Association with systemic lupus erythematosus in southwest Han Chinese. Ann Rheum Dis. 2003;62: 71–73. doi: 10.1136/ard.62.1.71 12480675 10.1136/ard.62.1.71 PMC1754297

[21] Senbagavalli P, Kumar N, Kaur G, Mehra NK, Geetha ST and Ramanathan VD. Major Histocompatibility Complex class III (C2, C4, factor B) and C3 gene variants in patients with pulmonary tuberculosis. Hum Immunol. 2011;72: 173–178. doi: 10.1016/j.humimm.2010.11.002 21093518 10.1016/j.humimm.2010.11.002

[22] Zhang S, Wang S, Li Q, Yao S, Zeng B, Ziegelstein RC, et al Capillary leak syndrome in children with C4A-deficiency undergoing cardiac surgery with cardiopulmonary bypass: A double-blind, randomised controlled study. Lancet. 2005;366: 556–562. doi: 10.1016/S0140-6736(05)67099-7 16099291 10.1016/S0140-6736(05)67099-7

[23] Szalai C, Fust G, Duba J, Kramer J, Romics L, Prohászka Z, et al Association of polymorphisms and allelic combinations in the tumour necrosis factor-alpha-complement MHC region with coronary artery disease. J Med Genet. 2002;39: 46–51. doi: 10.1136/jmg.39.1.46 11826025 10.1136/jmg.39.1.46 PMC1734954

[24] Jaatinen T, Ruuskanen O, Truedsson L, Lokki ML. Homozygous deletion of the CYP21a-TNXa-RP2-C4B gene region conferring C4B deficiency associated with recurrent respiratory infections. Hum Immunol. 1999;60: 707–714. 10439316 10.1016/s0198-8859(99)00047-6

[25] Soto K, Wu YL, Ortiz A, Aparicio SR, Yu CY. Familial C4B deficiency and immune complex glomerulonephritis. Clin Immunol. 2010;137: 166–175. doi: 10.1016/j.clim.2010.06.003 20580617 10.1016/j.clim.2010.06.003 PMC2941544

[26] Mougey R. A review of the Chido/Rodgers blood group. Immunohematology. 2010;26: 30–38. 20795316

[27] Samano ES, Ribeiro Lde M, Gorescu RG, Rocha KC, Grumach AS. Involvement of C4 allotypes in the pathogenesis of human diseases. Revista do Hospital das Clinicas. 2004;59: 138–144. 15286835 10.1590/s0041-87812004000300009

[28] Szilagyi A, Fust G. Diseases associated with the low copy number of the C4B gene encoding C4, the fourth component of complement. Cytogenetic and genome research. 2008;123: 118–130. doi: 10.1159/000184699 19287146 10.1159/000184699

[29] Margery-Muir AA, Wetherall JD, Castley AS, Hew M, Whidborne RS, Mallon DF, et al Establishment of gene copy number-specific normal ranges for serum C4 and its utility for interpretation in patients with chronically low serum C4 concentrations. Arthritis Rheumatol. 2014;66: 2512–2520. doi: 10.1002/art.38680 24757030 10.1002/art.38680

[30] Saxena K, Kitzmiller KJ, Wu YL, Zhou B, Esack N, Hiremath L, et al Great genotypic and phenotypic diversities associated with copy-number variations of complement C4 and RP-C4-CYP21-TNX (RCCX) modules: A comparison of Asian-Indian and European American populations. Mol Immunol. 2009;46: 1289–1303. doi: 10.1016/j.molimm.2008.11.018 19135723 10.1016/j.molimm.2008.11.018 PMC2716727

[31] Schwaeble WJ, Lynch NJ, Clark JE, Marber M, Samani NJ, Ali YM, et al Targeting of mannan-binding lectin-associated serine protease-2 confers protection from myocardial and gastrointestinal ischemia/reperfusion injury. Proc Natl Acad Sci U S A. 2011;108: 7523–7528. doi: 10.1073/pnas.1101748108 21502512 10.1073/pnas.1101748108 PMC3088599

[32] Klein J, Sato A. The HLA system. Second of two parts. N Engl J Med. 2000;343: 782–786. doi: 10.1056/NEJM200009143431106 10984567 10.1056/NEJM200009143431106

[33] Klein J, Sato A. The HLA system. First of two parts. N Engl J Med. 2000;343: 702–709. doi: 10.1056/NEJM200009073431006 10974135 10.1056/NEJM200009073431006

[34] Husby S, Koletzko S, Korponay-Szabo IR, Mearin ML, Phillips A, Shamir R, et al European society for pediatric gastroenterology, hepatology, and nutrition guidelines for the diagnosis of coeliac disease. J Pediatr Gastroenterol Nutr. 2012;54: 136–160. doi: 10.1097/MPG.0b013e31821a23d0 22197856 10.1097/MPG.0b013e31821a23d0

[35] Rittner C, DeMarchi M, Mollenhauer E, Carbonara A. Coeliac disease and C4A*q0: An association secondary to HLA-DR3. Tissue Antigens. 1984;23: 130–134. 6608806 10.1111/j.1399-0039.1984.tb00022.x

[36] Candore G, Lio D, Colonna Romano G, Caruso C. Pathogenesis of autoimmune diseases associated with 8.1 ancestral haplotype: Effect of multiple gene interactions. Autoimmun Rev. 2002;1: 29–35. 12849055 10.1016/s1568-9972(01)00004-0

[37] Grunewald J, Eklund A. Lofgren's syndrome: Human Leukocyte Antigen strongly influences the disease course. Am J Respir Crit Care Med. 2009;179: 307–312. doi: 10.1164/rccm.200807-1082OC 18996998 10.1164/rccm.200807-1082OC

[38] Wennerstrom A, Pietinalho A, Vauhkonen H, Lahtela L, Palikhe A, Herman J, et al HLA-DRb1 allele frequencies and C4 copy number variation in Finnish sarcoidosis patients and associations with disease prognosis. Hum Immunol. 2012;73: 93–100. doi: 10.1016/j.humimm.2011.10.016 22074998 10.1016/j.humimm.2011.10.016

[39] Kainulainen L, Peltola V, Seppanen M, Viander M, He Q, Lokki ML, et al C4A deficiency in children and adolescents with recurrent respiratory infections. Hum Immunol. 2012;73: 498–501. doi: 10.1016/j.humimm.2012.02.015 22406254 10.1016/j.humimm.2012.02.015

[40] Sane J, Kurkela S, Lokki ML, Miettinen A, Helve T, Vaheri A, et al Clinical sindbis alphavirus infection is associated with HLA-DRB1*01 allele and production of autoantibodies. Clinical infectious diseases: an official publication of the Infectious Diseases Society of America. 2012;55: 358–363. 10.1093/cid/cis405 22523259

[41] Skattum L, van Deuren M, van der Poll T, Truedsson L. Complement deficiency states and associated infections. Mol Immunol. 2011;48: 1643–1655. doi: 10.1016/j.molimm.2011.05.001 21624663 10.1016/j.molimm.2011.05.001

[42] Mostafa GA, Shehab AA. The link of C4B null allele to autism and to a family history of autoimmunity in Egyptian autistic children. J Neuroimmunol. 2010;223: 115–119. doi: 10.1016/j.jneuroim.2010.03.025 20452682 10.1016/j.jneuroim.2010.03.025

[43] Rigby WF, Wu YL, Zan M, Zhou B, Rosengren S, Carlson C, et al Increased frequency of complement C4B deficiency in rheumatoid arthritis. Arthritis Rheum. 2012;64: 1338–1344. doi: 10.1002/art.33472 22076784 10.1002/art.33472 PMC3775471

