# Supplementary material for: Clinical features of patients with homozygous complement C4A or C4B deficiency
Source: PLoS One. 2018 Jun 21;13(6):e0199305. doi: 10.1371/journal.pone.0199305 (PMC6013154; doi:10.1371/journal.pone.0199305)
Supplement: S1 Table — (DOCX) [file pone.0199305.s001.docx]

| **S1 Table. Post-infectious symptoms in 20 patients with complete C4B deficiency** | |
| --- | --- |
| **Preceding infection** | **Clinical symptoms** |
| *Herpes virus 2 (n=1)* | Prolonged mucous ulcers |
| *Varicella zoster virus (n=1)* | Neurological symptoms |
| Streptococcal disease (n=2) | Relapsing myopericarditis (n=1), arthralgia (n=1) |
| Tuberculosis (n=3) | cutaneous vasculitis (n=1), erythema induratum (n=1), immune complex disease (n=1) |
| Staphylococcus aureus (n=1) | Reactive arthritis (n=1) |
| Vaccination (n=2) | Guillain-Barre syndrome (n=1), prolonged fever (n=1) |
| *Enterovirus (n=1)* | sicca (n=1) |
| Unspecified respiratory infection (n=6) | Relapsing myopericarditis (n=1), muscle and joint pain (n=3), SLE like disease (n=1), uveitis (n=1) |
| Gastroenteritis (n=3) | opticus neuritis (n=1), proximal muscle weakness with liquor pleocytosis (n=1), erythema induratum (n=1) |
