# Supplementary material for: Clinical features of patients with homozygous complement C4A or C4B deficiency
Source: PLoS One. 2018 Jun 21;13(6):e0199305. doi: 10.1371/journal.pone.0199305 (PMC6013154; doi:10.1371/journal.pone.0199305)
Supplement: S2 Table — (DOCX) [file pone.0199305.s002.docx]

| **S2 Table. Available complement activities in study populations** | | | | | |
| --- | --- | --- | --- | --- | --- |
|  | **C4A def** | **C4B def** | **Controls** | **C4A def vs. Controls p=** | **C4Bdef vs Controls p=** |
| Complement study | **(n=9)** | **(n=23)** | **(n=32)** |  |  |
| Classical pathway activity (%) | 110 (91) | 109 (43) | 103 (95) | 0.489 | 0.937 |
| Alternative pathway activity (%) | 100 (49) | 96 (148) | 99 (97) | 0.156 | 0.325 |
| Lectin pathway activity (%) | 49 (138) | 59 (172) | 106 (169) | 0.176 | 0.008 |
| Lowered Classical pathway activity (<75% | 1 (11.1) | 0 (0) | 2 (6.3) | 0.535 | 0.504 |
| Lowered alternative pathway activity (<40%) | 0 (0) | 0 (0) | 1 (3.2) | 1.000 | 1.000 |
| Lowered lectin pathway activity (<11%) | 0 (0) | 8 (34.8) | 3 (9.4) | 1.000 | 0.038* |
| Percentage of the initial study population | 28.1 | 26.4 | 26.7 |  |  |
| Data are expressed as n(%) or as median (range), as appropriate | | |  |  |  |
| * CI=5.16, 95%CI=1.19–22.33 |  |  |  |  |  |
