# Supplementary material for: Clinical features of patients with homozygous complement C4A or C4B deficiency
Source: PLoS One. 2018 Jun 21;13(6):e0199305. doi: 10.1371/journal.pone.0199305 (PMC6013154; doi:10.1371/journal.pone.0199305)
Supplement: S3 Table — (DOCX) [file pone.0199305.s003.docx]

| **S3 Table. Number of C4 genes in study populations** | | | |
| --- | --- | --- | --- |
| **Number of C4A genes** | **Total C4A deficiency** | **Total C4B deficiency** | **Controls** |
| 0 | 32 (100) | 0 (0) | 0 (0) |
| 2 | 0 (0) | 21 (24.1) | 120 (100) |
| 3 | 0 (0) | 60 (67.0) | 0 (0) |
| 4 | 0 (0) | 6 (7.0) | 0 (0) |
| **Number of C4B genes** |  |  |  |
| 0 | 0 (0) | 87 (100) | 0 (0) |
| 2 | 30 (93.8) | 0 (0) | 120 (100) |
| 3 | 2 (6.3) | 0 (0) | 0 (0) |
| Data expressed as n (%) | | | |
